# Skill-File-Guided Virtual Screening: A Single-Target Formyl Peptide Receptor 2 Case Study of Large-Language-Model-Coordinated Physics and Diffusion Docking

**DOI:** 10.34133/csbj.0187

**Published:** 2026-09-09

**Authors:** Osman Gani

**Affiliations:** Section for Pharmaceutical Chemistry, Department of Pharmacy, University of Oslo, 0316 Oslo, Norway.

## Abstract

**Background:** Virtual screening outcomes depend on protocol choices normally made implicitly by experts. I tested whether encoding that expertise as a “skill file” (a Markdown reference read by a large-language-model coding agent) changes virtual screening outcomes versus naive agent defaults, in a single-target case study on formyl peptide receptor 2 (FPR2; Protein Data Bank 7T6S, 3.0 Å cryo-electron microscopy). **Methods:** A Claude Code agent curated 1,000 pChEMBL ≥5 FPR2 actives and 9,829 property-matched decoys, prepared ligand libraries under naive and skill-guided protocols, and screened them with 2 engines: Uni-Dock (graphics-processing-unit-accelerated AutoDock Vina) and DiffDock v1 (native confidence score). The evaluation used receiver operating characteristic (ROC) area under the curve (AUC), Boltzmann-enhanced discrimination of ROC, enrichment factors (EFs), and paired bias-corrected and accelerated bootstrap, DeLong, and permutation tests. **Results:** On Uni-Dock, the skill-guided protocol raised ROC AUC from 0.701 to 0.733 (paired ΔAUC = +0.020; DeLong *P* = 3.4 × 10^−3^), while top-of-list enrichment regressed (EF@1% 2.67 → 1.23; EF@0.5% 3.33 → 0.00). Because the arms differ on 5 axes at once, this is a joint-protocol effect, not skill-file guidance alone. DiffDock v1 stayed near random (AUC 0.54 to 0.56) on this out-of-distribution G protein-coupled receptor. **Conclusion:** In this single-target case study, skill-file-guided protocol selection improved global ranking for a physics-based engine on FPR2 while degrading early enrichment, and it did not rescue a diffusion model outside its training distribution. The +0.020 AUC gain reflects a bundled preparation-and-docking-parameter intervention rather than the skill file alone, comes from a single run per protocol with no seed control, and awaits multitarget, multi-seed replication. Code, data, and the skill file are openly released.

## Introduction

Structure-based virtual screening (VS) is widely used in computational drug discovery to prioritize candidate molecules for experimental testing from large chemical libraries [1,2]. Performance depends on protocol decisions at every stage: receptor preparation, binding-site definition, ligand library curation, docking engine selection, search exhaustiveness, and post-docking analysis [3]. These decisions are normally taken by experienced computational chemists and are rarely evaluated systematically, which limits reproducibility.

Docking engines fall into 2 broad classes. Physics-based methods, exemplified by AutoDock Vina [4] and its graphics processing unit (GPU)-accelerated derivative Uni-Dock [5], use empirically parameterized scoring functions combining van der Waals, electrostatic, hydrogen bonding, and desolvation terms; they are interpretable, transferable across target classes, and fast enough that Uni-Dock can process more than 1 million compounds per day on a single GPU [5]. Machine learning (ML) methods, led by DiffDock [6], instead learn to predict binding poses from training data using diffusion generative models over the product space of translations, rotations, and torsion angles. DiffDock was reported to achieve state-of-the-art blind docking performance [6], but subsequent independent benchmarks have raised concerns about training set memorization [7], physical validity of generated poses [8], and poor generalization to targets outside the training distribution [9].

G protein-coupled receptors (GPCRs) are a challenging test case for ML-based docking. GPCRs are severely under-represented in PDBBind, the standard training set for ML docking methods, because most GPCR structures were solved by cryo-electron microscopy (cryo-EM) after 2019, which is outside DiffDock’s training window [10]. Furthermore, cryo-EM structures at 3- to 5-Å resolution introduce coordinate uncertainty that degrades all docking methods [11]. That DiffDock generalizes poorly to out-of-distribution targets is already established [7–9]; what remains untested is whether this generalization gap is large enough to be practically decisive in a VS setting, i.e., whether it persists, and at what magnitude, when DiffDock is benchmarked head-to-head against a physics-based engine on enrichment metrics for a single GPCR VS task, rather than on pose-recovery metrics alone.

In parallel, large language model (LLM) coding agents are increasingly used to automate scientific workflows. Coscientist [12] and ChemCrow [13] have already shown that an LLM agent can orchestrate multistep chemistry tasks, including database queries, code generation, and result analysis, so the general capability of LLM agents to run scientific pipelines is established, not novel [12–14]. What has not been tested is a narrower and more specific question: whether supplying such an agent with a structured, human-authored reference document, a “skill file”, changes the quality of the protocol it produces relative to letting it rely on tool defaults and its own judgment. A skill file in this sense is a static Markdown document of expert procedural knowledge that the agent consults during planning, before it issues any tool call for that stage of the pipeline, and that it may consult repeatedly but cannot itself edit during the run. This differs mechanistically from a system prompt, which sets persistent behavioral constraints applied uniformly across the whole session rather than stage-specific domain procedure; from retrieval-augmented generation, which retrieves passages dynamically from a corpus at query time rather than presenting a single fixed reference the agent returns to; and from a fine-tuned model, which bakes the knowledge into model weights rather than leaving it as an externally editable, swappable document. We are not aware of a published benchmark that isolates this specific mechanism, a single static procedural reference document consulted at a fixed point in the agent loop, as a controllable input to an agent-driven VS pipeline and measures its effect on docking-protocol quality; this absence has not been confirmed by a systematic literature search and should be read as a gap we identified, not an exhaustively verified claim.

In this study, I therefore asked whether giving an LLM coding agent (Claude Code [15]) access to such a skill file changes the quality of the VS protocol it produces and whether any such effect depends on the docking engine the agent is directing.

I used the agent to design, implement, and execute a VS benchmark pipeline under close human scientific supervision, requiring only high-level scientific direction (e.g., “dock these against FPR2, compare Uni-Dock vs DiffDock”); this execution capability is the substrate for the skill-file contrast, not a contribution in its own right, since Coscientist [12] and ChemCrow [13] already established that LLM agents can run multistep scientific pipelines. The primary contribution is this skill-file contrast: Identical high-level instructions were given to the agent with and without access to the skill file, and the resulting docking protocols and VS performance were compared for formyl peptide receptor 2 (FPR2), a GPCR with a 3.0-Å cryo-EM structure (Protein Data Bank (PDB): 7T6S [16]). Because the with- and without-skill runs differ as complete protocols rather than on the skill-file mechanism alone, the study is best read as a test of whether expert procedural guidance, delivered through a skill file, improves an agent-driven VS workflow relative to tool defaults, not as an isolation of the skill-file mechanism in itself. As a secondary, exploratory axis of the same benchmark, the 2 protocols were each run with a physics-based engine (Uni-Dock [5]) and an ML engine (DiffDock [6]), allowing a within-study comparison of whether the skill file’s effect depends on the docking engine. The comparison reported below is a single-target case study on FPR2, not a multitarget benchmark; generalization across additional targets and protein families is left to future work.

## Methods

### Agent setup and workflow

All computational work was performed by Claude Code (Anthropic, model claude-opus-4-8 [15]), an LLM-based coding agent operating in a Linux terminal environment with access to file system operations, shell commands, and Python/conda package management. The runs were carried out on an Ubuntu 24.04 LTS workstation (kernel 6.17.0-oem) over the period 1 March 2026 to 13 March 2026 (analysis outputs finalized 13 March 2026); the Claude Code CLI used was version 2.1.197. The full agent transcript, the prompts issued, and the Model Context Protocol (MCP)/plugin configuration are deposited in the Supplementary Material’s “Agent execution log” subsection and in the Zenodo record [17]. The agent received natural-language instructions describing the scientific objective but was not provided with pre-written scripts. Two parallel runs were conducted: a “naive” protocol where the agent used tool defaults and a “skill-guided” protocol where the agent had access to a structured reference document (skill file) encoding expert best practices for VS [17]. The skill file was authored before any FPR2 scores were computed; no parameter was tuned on the held-out evaluation. The pipeline was executed once per protocol; no repeated runs were performed to characterize agent-to-agent or seed-to-seed variance for this single-target study (see “Limitations” section).

The skill file is a Markdown document (~200 lines) specifying a 7-stage VS pipeline: target preparation, binding-site definition, library preparation [including pan-assay interference compounds (PAINS) [18] and Brenk [19] filters, molecular weight 150 to 600 Da, ALogP −2 to 5], docking parameters (box size, 25 Å; exhaustiveness, 32), post-processing, hit triage, and validation. The exhaustiveness and box-size recommendations reflect general best practice for AutoDock-Vina-class scoring [4,5], not a value tuned on this target. The agent consulted this file when making protocol decisions in the skill-guided arm. The exact version of the skill file used here is deposited as SKILL.md in the Zenodo record [17].

### Target system

FPR2 (ChEMBL ID CHEMBL4227) was selected as the target. The receptor structure was obtained from PDB entry 7T6S [16], a 3.0-Å cryo-EM structure of FPR2 in complex with compound C43 (FUI). 7T6S is a multichain deposit (receptor plus heterotrimeric G-protein and a stabilizing nanobody); chain R, the FPR2 receptor, was extracted and used for docking; all other chains, crystallographic waters, and ions were removed. Receptor preparation used Meeko mk_prepare_receptor at physiological pH with default protonation-state assignment (no explicit overrides). The docking box was centered on the co-crystallized ligand FUI at coordinates *x* = 81.893, *y* = 115.414, and *z* = 96.832 Å, with per-axis dimensions 20 × 20 × 20 Å in the naive protocol and 25 × 25 × 25 Å in the skill-guided protocol (deposited as 05_docking_v2/unidock_naive/config.txt and 05_docking_v2/unidock_skill/config.txt in the Zenodo record [17]). The co-crystallized ligand FUI served as the reference for binding-site definition and redocking validation.

### Active compound curation

The agent queried ChEMBL (release 36 [20]) via the chembl_webresource_client Python API in March 2026 for FPR2 bioactivity data. The activity filter was target_chembl_id = CHEMBL4227, pchembl_value ≥ 5, standard_relation = "=" (excluding qualified inequality values), and standard_type in {EC_50_, IC_50_, *K*_i_, *K*_d_} (half-maximal effective concentration, half-maximal inhibitory concentration, inhibition constant, and dissociation constant, respectively). Curation included Simplified Molecular Input Line Entry System standardization with RDKit [21] (the exact version is pinned in the deposited environment_vina.yml conda-environment file in the Zenodo record [17]), removal of salts and duplicates (InChIKey connectivity layer), and removal of compounds with extreme properties (molecular weight <150 or >600 Da). When a compound carried multiple activity records, the highest pChEMBL value was retained. Butina clustering (Tanimoto distance cutoff 0.4 on Morgan fingerprints, radius 2 [22]) was applied to ensure structural diversity, followed by MaxMin diversity picking on the cluster representatives. The final set comprised 1,000 diverse active compounds, deposited as 01_active_curation_v2/actives_curated_v2.csv (library = 1,000 actives + 9,829 decoys = 10,829 total; confirmed as the sum of the deposited actives and decoy sets).

### Decoy generation

Property-matched decoys were generated from a drug-like subset of ChEMBL following the DUD-E (directory of useful decoys, enhanced) principles [23] (ChEMBL was used in place of ZINC so the actives and decoys are drawn from a comparable bioactivity-screened library). For each active, decoys were selected to match molecular weight (±25 Da), calculated LogP (±1.0), number of hydrogen-bond donors (±1), hydrogen-bond acceptors (±2), and rotatable bonds (±2), while maintaining a Tanimoto coefficient <0.35 to the nearest active. Tanimoto similarity used Morgan fingerprints (radius 2, 2048-bit; equivalent to the extended-connectivity fingerprint of diameter 4). Any compound with an FPR2 bioactivity record in ChEMBL (target_chembl_id = CHEMBL4227) was excluded from the decoy pool, irrespective of measured value, to remove putative true binders from the negative class. When the tolerance window yielded no matching pool compound for a given active, that active was skipped at the matching step and the pool sampling moved to the next active; the random seed for pool sampling was 42 (generate_decoys_local.py). This produced 9,829 decoys (approximately 10:1 ratio; library total 10,829 = 1,000 actives + 9,829 decoys). Property-matching quality was verified by Kolmogorov–Smirnov tests on all 5 property distributions, and Tanimoto separation was independently verified (02_decoy_generation_v2/qc_tanimoto_separation.py in the Zenodo record). Decoy compound IDs are deposited as 02_decoy_generation_v2/decoys_curated.csv. Formal charge was not matched explicitly; hidden correlations beyond the 5 matched properties (e.g., aromatic ring count and scaffold complexity) were not audited and are noted as a limitation of the decoy design.

### Library preparation

Two preparation protocols were applied in parallel (Table 1):

**Table 1.** Protocol comparison between naive and skill-guided arms

Parameter	Naive	Skill-guided
Receptor preparation	OpenBabel	Meeko mk_prepare_receptor
Ligand 3D generation	OpenBabel gen3d	RDKit ETKDGv3
Ligand PDBQT conversion	OpenBabel	Meeko mk_prepare_ligand
Chemical filters	None	PAINS + Brenk (RDKit)
Docking box (Uni-Dock)	20 Å	25 Å
Exhaustiveness (Uni-Dock)	8 (default)	32 (recommended)
DiffDock inference steps	20 (default)	25
DiffDock samples per complex	10 (default)	20
DiffDock batch size	8	4
DiffDock ESM embeddings	Computed inline	Pre-computed before docking
Input library size	10,829 compounds (1,000 actives + 9,829 decoys)	5,240 compounds after PAINS/Brenk filtering (5,589 removed)

PAINS, pan-assay interference compounds; PDBQT, Protein Data Bank with partial charges and atom types; ESM, evolutionary scale modeling

The input library (10,829 compounds: 1,000 actives and 9,829 decoys) is identical for naive and skill-guided arms before filtering; the skill-guided arm’s PAINS/Brenk filter removes 5,589 compounds, leaving 5,240 (03_library_preparation_v2/skill/filter_report_v2.csv in the Zenodo record [17]). Because PAINS/Brenk filtering is not class-blind, it removes actives and decoys at different rates: of the 1,000 curated actives, 427 (43%) fail the filter, leaving 573 (DiffDock arm) or 570 (Uni-Dock arm) skill-guided actives; the small 573-versus-570 difference reflects per-engine ligand-preparation attrition on the same filtered input list, not a separate filtering step. The naive and skill-guided arms are therefore scored on different, nonequivalent active sets (chemotype-restricted in the skill arm); see “Limitations” section for the implications for the area under the curve (AUC) comparison. Per-engine scored compound counts (after ligand-preparation failures) are reconciled in data/metrics_all.csv: Uni-Dock-naive 9,519 (600 actives + 8,919 decoys), Uni-Dock-skill 4,865 (570 actives + 4,295 decoys), DiffDock-naive 9,858 (913 actives + 8,945 decoys), and DiffDock-skill 5,201 (573 actives + 4,628 decoys). A Consolidated Standards of Reporting Trials-style compound-flow table reconstructing this attrition end-to-end (curated → filtered → prepared → scored, per arm and per engine) is included as Table S5.

The attrition has 3 distinct origins. In the skill-guided arm, it is dominated by the intentional PAINS/Brenk filter, which removes 5,589 of 10,829 compounds (51.6%; the most frequent flags are Brenk aliphatic long chains, *n* = 1,188; imines, *n* = 493; isolated alkenes, *n* = 447; Michael acceptors, *n* = 243; and alkyl halides, *n* = 203). A further 17 compounds (0.3% of the 5,240 that pass the filter) fail RDKit/Meeko ligand preparation, and the remaining loss occurs at the docking step (Uni-Dock skill-guided: 358 of 5,223 prepared, 6.9%; DiffDock skill-guided: 22 of 5,223, 0.4%). The naive arm applies no chemical filter, yet the Uni-Dock naive arm still scores only 600 of 1,000 actives and 8,919 of 9,829 decoys; this approximately 40% active attrition arises from OpenBabel 3D-generation and Protein Data Bank with partial charges and atom types (PDBQT)-conversion failures together with Uni-Dock batch-mode crashes (the raw docking logs record batch segmentation faults), but the deposited naive preparation log is not machine-readable, so the exact split between preparation and docking failures for that arm cannot be tabulated and only the totals are reported. DiffDock naive, by contrast, scored 913 of 1,000 actives (8.7% loss). The full per-arm, per-engine compound-flow cascade (curated → filtered → prepared → scored) is given in Table S5.

### Ligand protonation, tautomers, and conformers

Ligand protonation and tautomer handling differed from the receptor procedure and are reported here explicitly since both can influence GPCR docking. In the naive arm, ligands were processed with OpenBabel (obabel --gen3d -h, Gasteiger partial charges), producing a single 3D conformer per ligand with hydrogens added for the neutral, valence-based state; no pH-specific protonation model and no tautomer enumeration were applied. In the skill-guided arm, ligands were standardized and salt-stripped with RDKit, protonated with RDKit AddHs, embedded with Experimental-Torsion Knowledge Distance Geometry version 3 (ETKDGv3; a single conformer per ligand, random seed 42), minimized with the Merck molecular force field (MMFF, with the universal force field, UFF, as fallback), and converted to PDBQT with Meeko; again, no pH-targeted protonation model (e.g., Dimorphite-DL or Epik) and no tautomer enumeration were used. DiffDock received Simplified Molecular Input Line Entry System input (raw in the naive arm, RDKit canonical in the skill-guided arm) and assigned protonation internally. The skill file itself recommends assigning protonation states at pH 7.4 and enumerating tautomers, but these 2 steps were not implemented in the executed pipeline; ligand protonation was therefore handled by tool defaults in both arms and is not a differentiating axis between them. pH-aware protonation and tautomer enumeration are a concrete refinement for future runs.

### Docking engines

Uni-Dock (v1.1.3 [5]) is a GPU-accelerated implementation of the AutoDock Vina scoring function. Ligands were docked in batches of 200 using the Uni-Dock command-line interface. The Uni-Dock --seed flag was not explicitly set; the tool default was used. Scores were extracted as the most negative VINA RESULT energy (kcal/mol) per compound; more negative values indicate stronger predicted binding. The per-arm Uni-Dock configuration files are deposited in 05_docking_v2/unidock_naive/config.txt and 05_docking_v2/unidock_skill/config.txt in the Zenodo record [17].

DiffDock [6] is a diffusion generative model that predicts protein–ligand binding poses by learning to reverse a diffusion process over rigid-body and torsional degrees of freedom. The score-model checkpoint at workdir/v1.1/score_model in the public DiffDock distribution was used (DiffDock v1.1, the version paired with the original DiffDock paper [6]). Inference was blind, using the default DiffDock inference.py configuration file default_inference_args.yaml in the naive arm and skill_inference_args.yaml in the skill-guided arm (both deposited in the Zenodo record [17]). The skill-guided arm differed from the naive arm in 3 respects: the number of denoising steps was raised from 20 (default) to 25, the number of samples per complex from 10 to 20, and the per-protein evolutionary scale modeling (ESM) embeddings were pre-computed and supplied via the --esm_embeddings_path flag rather than being recomputed inline at each call; the batch size was reduced from 8 to 4 to accommodate the larger sample count within GPU memory. The torch random seed was not explicitly set in either arm; the DiffDock-default seeding was used. The top-ranked pose’s confidence score was used for VS ranking; higher confidence indicates greater predicted reliability.

### Single-ligand redocking validation

Before the VS campaign, the co-crystallized ligand FUI was redocked into FPR2 using 3 engines: AutoDock Vina 1.2 (central processing unit), Uni-Dock (GPU batch mode), and DiffDock (Table 2). Heavy-atom root-mean-square deviation (RMSD) to the crystal pose was computed for the top-ranked prediction. Success was defined as the conventional RMSD <2.0 Å cutoff used in pose-prediction and comparative assessment of scoring functions-style docking benchmarks (see e.g. [24,25]).

**Table 2.** Single-ligand redocking of FUI into FPR2 (PDB: 7T6S)

Engine	Protocol	RMSD (Å)	Success	Notes
Vina 1.2	Naive	0.22	Yes	
Vina 1.2	Skill	0.39	Yes	
Uni-Dock	Naive	5.18	No	Batch mode
Uni-Dock	Skill	5.68	No	Batch mode
DiffDock	Naive	23.4	No	Conf. −0.86
DiffDock	Skill	28.7	No	Conf. −0.92

RMSD, root-mean-square deviation

### Evaluation metrics

VS performance was assessed using receiver operating characteristic (ROC) area under the curve (AUC) [26], Boltzmann-enhanced discrimination of ROC (BEDROC) (*α* = 20 and 80.5 [27]), enrichment factors (EFs) at 0.5%, 1%, 5%, and 10%, LogAUC [28], area under the precision–recall curve, and net reclassification improvement/integrated discrimination improvement (NRI/IDI) between the naive and skill-guided score distributions for each engine, computed only on the subset of compounds scored by both protocols within an engine (paired Uni-Dock *n* = 4,303; paired DiffDock *n* = 4,765; see below for class composition). For readers less familiar with these metrics, ROC AUC is the probability that a randomly chosen active is ranked above a randomly chosen decoy (0.5 indicates random ranking; 1.0, perfect ranking); the EF at x% (EF@x%) is the fold-enrichment in actives among the top x% of the ranked library relative to a random selection; and BEDROC is an early-enrichment-weighted enrichment measure (here with *α* = 20) bounded to [0,1], for which Truchon and Bayly [27] give the formal definition. Continuous NRI used the sign of the within-compound score change (threshold-free); IDI used min–max-normalized per-arm scores as pseudo-probabilities, which puts AutoDock Vina kcal/mol energies and DiffDock confidence scores on a common [0,1] scale within each engine without requiring a Platt/isotonic calibration model. Cliff’s *d* was computed as an unpaired rank-biserial effect-size complement to AUC (*d* = 2·AUC − 1 for the unpaired case).

Results are reported separately for each arm’s own scored compound set (Table 3); no cross-arm imputation onto a common ground truth was performed in the version of the pipeline whose outputs are deposited in data/metrics_all.csv because the naive and skill-guided arms do not share an identical active set after PAINS/Brenk filtering (see “Library preparation” section). The paired comparisons reported in the “Results” and “Discussion” sections use the intersection of compounds scored by both protocols within a given engine (paired Uni-Dock *n* = 4,303; paired DiffDock *n* = 4,765).

**Table 3.** Virtual screening enrichment metrics for all 4 protocols

Metric	Uni-Dock naive	Uni-Dock skill	DiffDock naive	DiffDock skill
*n* (actives/decoys)	9,519 (600/8,919)	4,865 (570/4,295)	9,858 (913/8,945)	5,201 (573/4,628)
ROC AUC	0.701	0.733	0.538	0.564
95% CI (BCa)	0.683 to 0.719	0.713 to 0.752	0.520 to 0.557	0.542 to 0.586
Permutation *P*	≤1.0 × 10^−4^	≤1.0 × 10^−4^	≤1.0 × 10^−4^	≤1.0 × 10^−4^
BEDROC (*α* = 20)	0.163	0.258	0.095	0.115
EF 1%	2.67	1.23	0.77	0.87
EF 5%	1.83	1.86	0.74	0.91
EF 10%	1.78	2.11	0.91	0.84
LogAUC	0.232	0.248	0.151	0.157
AUPRC	0.112	0.217	0.097	0.120

ROC, receiver operating characteristic; AUC, area under the curve; BEDROC, Boltzmann-enhanced discrimination of ROC; EF, enrichment factor; AUPRC, area under the precision–recall curve; CI, confidence interval; BCa, bias-corrected and accelerated

Standard errors were computed by the Hanley–McNeil method [26]. Confidence intervals (CIs) were obtained by bias-corrected and accelerated (BCa) bootstrap with 10,000 iterations and a fixed random seed (SEED = 42, numpy.random.RandomState) [29]. Bootstrap resampling drew the same row index for both arms of a paired comparison (case resampling with shared indices), so paired bootstraps are paired by compound; resampling was not separately stratified by class (active/decoy), only checked post hoc to exclude resamples containing zero actives or zero decoys. For the paired Uni-Dock comparison (*n* = 4,303 compounds scored by both protocols), the intersection set’s class composition recovered from the NRI/IDI event counts is approximately 340 actives and 3,969 decoys; for paired DiffDock (*n* = 4,765), approximately 533 actives and 4,258 decoys (these counts sum to 4,309 and 4,791, which differ from the Wilcoxon-reported n_pairs of 4,303 and 4,765 by 6 and 26 compounds, respectively, reflecting tied-score exclusions in the Wilcoxon step that the NRI/IDI implementation does not apply). Pairwise AUC comparisons used the DeLong test [30] with Benjamini–Hochberg correction for multiple testing [31] applied across all 6 pairwise protocol comparisons among the 4 protocols (unidock_naive, unidock_skill, diffdock_naive, and diffdock_skill); family size *m* = 6, consistent with multiple_testing_correction_delong in data/statistical_analysis_v2.json. The headline claims in the “Results and Discussion” sections use only the 2 within-engine paired comparisons (Uni-Dock skill versus naive, *P* = 3.4 × 10^−3^; DiffDock skill versus naive, *P* = 0.63); the 4 cross-engine DeLong comparisons, whose asymptotic-normal-approximation *P* values fall well below 10^−40^ on the deposited script, are not relied on for any headline claim. Permutation tests (10,000 iterations, same fixed seed) assessed whether each protocol’s AUC exceeded chance (0.5); the smallest reportable permutation *P* value at this iteration count is the floor 1/(*B* + 1) = 1/(10,000 + 1) ≈ 1.0 × 10^−4^, so each permutation *P* in the “Results and Discussion” sections is reported as *P* ≤ 1.0 × 10^−4^ at this bound, not as a strict inequality below it.

### Computational resources

All experiments were performed on a workstation equipped with 2 NVIDIA RTX 4500 Ada GPUs (24 GB VRAM each) and CUDA 12.9. The analysis conda environment (vina_dock, Python 3.12) included AutoDock Vina, Meeko 0.7.1, RDKit, OpenBabel, SciPy, NumPy, BioPython, and scikit-learn; DiffDock was installed in a separate conda environment (diffdock, Python 3.9, CUDA 12.1). The exact version pins for both environments are recorded in environment_vina.yml and environment_diffdock.yml in the Zenodo record [17].

## Results

### Agent-driven pipeline execution

**Fig. 1.**
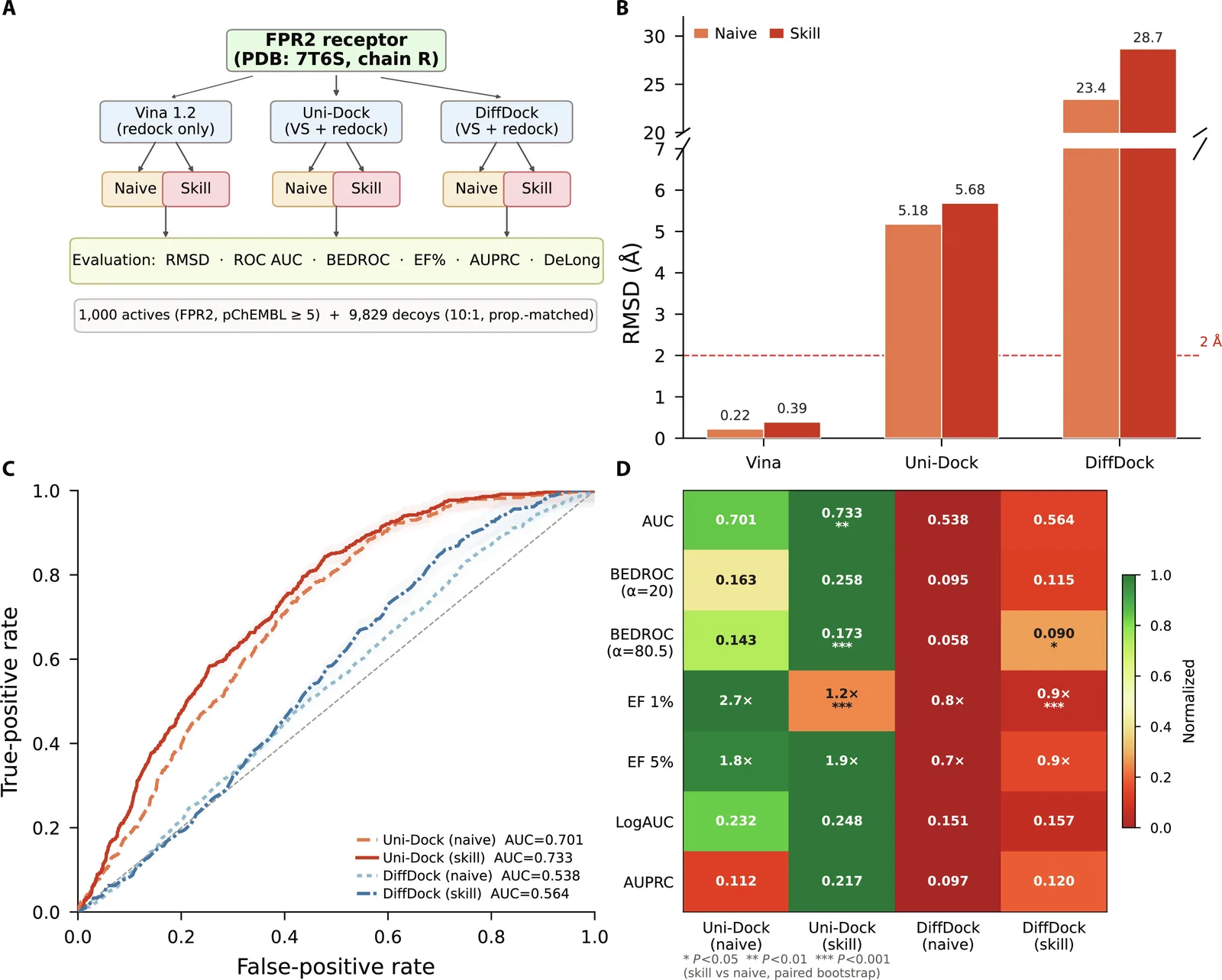
Study design and headline virtual-screening results. (A) Study design: the formyl peptide receptor 2 (FPR2) [Protein Data Bank (PDB) 7T6S, chain R] was screened against 1,000 pChEMBL ≥5 actives and 9,829 property-matched decoys under 3 engine configurations (AutoDock Vina 1.2 for redocking only; Uni-Dock and DiffDock for both virtual screening and redocking), each run in a naive and a skill-guided arm. Evaluation used root-mean-square deviation (RMSD), receiver operating characteristic (ROC) area under the curve (AUC), Boltzmann-enhanced discrimination of ROC (BEDROC), enrichment factor (EF), area under the precision–recall curve (AUPRC), and DeLong tests. (B) Single-ligand redocking RMSD of the co-crystallized ligand FUI into 7T6S for each engine; the 2-Å redocking-success threshold is marked. (C) Full-range ROC curves for all 4 VS protocols, with the legend reporting per-arm AUC values. (D) Heatmap of the per-metric performance summarized in Table 3, with cell color showing each metric min–max normalized across the 4 protocols (0 = lowest; 1 = highest), raw metric values overlaid as text, and paired-bootstrap significance (skill versus naive) indicated by asterisks. Low-FPR/early-enrichment behavior is summarized by LogAUC and by BEDROC (*α* = 20, *α* = 80.5) rows in this panel, and quantitatively by the EF@1% and EF@5% rows.

The agent executed every stage of the pipeline without human coding intervention (Figure 1). Starting from a single natural-language instruction (“benchmark Uni-Dock vs DiffDock on FPR2 with 1,000 actives and property-matched decoys”), the agent (a) queried ChEMBL via the Python API and curated 1,000 actives with diversity selection; (b) generated 9,829 property-matched decoys with QC validation; (c) prepared PDBQT and structure-data file libraries for both protocols; (d) configured and launched docking jobs on 2 GPUs; (e) parsed output files and extracted scores; (f) computed enrichment metrics, bootstrap CIs, and DeLong tests; (g) generated publication-quality figures. The pipeline required approximately 182 GPU-hours for DiffDock and 13 GPU-hours for Uni-Dock.

### Dataset characteristics

The curated library comprised 1,000 FPR2 actives (median pChEMBL = 6.1, interquartile range 5.4 to 7.0) and 9,829 property-matched decoys. Kolmogorov–Smirnov tests confirmed no significant differences in molecular weight, LogP, hydrogen-bond donors, hydrogen-bond acceptors, or rotatable bond distributions between actives and decoys (all *P* > 0.05). The skill-guided protocol filtered the library to 5,240 compounds (removing 5,589 via PAINS/Brenk filters), of which 573 were actives (427 of the 1,000 curated actives, 43%, removed by filtering). Because PAINS/Brenk filtering was applied only in the skill-guided arm, the skill-arm AUC in Table 3 is computed on a smaller, chemotype-restricted active set, not the same ground truth used for the naive arm; this is a potential source of the observed AUC difference independent of docking-parameter changes (see “Discussion” section).

### Single-ligand redocking

Only Vina successfully reproduced the crystal pose (RMSD < 2.0 Å; Table 2). Uni-Dock in batch mode placed the ligand outside the orthosteric pocket (RMSD 5.2 to 5.7 Å). This poor single-ligand recovery is unlikely to have a single cause: Batch-mode preparation with a large search volume, receptor preparation and binding-site definition, and ligand protonation or tautomer assignment can each degrade redocking accuracy, and we do not isolate their relative contributions here. DiffDock predictions were effectively random (RMSD 23 to 29 Å), with negative confidence scores indicating the model’s own assessment of poor reliability.

### VS performance

Uni-Dock achieved meaningful VS enrichment with both protocols (Table 3). The skill-guided protocol significantly outperformed naive defaults on global ranking discrimination (ROC AUC 0.733 versus 0.701; ΔAUC = +0.020 on paired compounds, *n* = 4,303 compounds scored by both protocols; DeLong *P* = 0.003; paired bootstrap 95% CI 0.007 to 0.034, 2-sided *P* = 0.002). This improvement was not uniform across the ranked list: EF at the top of the list moved in the opposite direction, falling from 2.67 to 1.23 at the 1% threshold and from 3.33 to 0.00 at the 0.5% threshold (paired Δ = −3.24, 95% CI −4.94 to −1.19), while EF at 5% and 10% improved modestly (1.83 → 1.86 and 1.78 → 2.11). The skill-guided protocol therefore improved overall ranking discrimination and mid-list enrichment while degrading enrichment among the very top-ranked candidates. Reclassification analysis confirmed this trade-off: The integrated discrimination improvement was negative (IDI = −0.0398, *z* = −3.10, *P* = 0.0019), indicating that, net of direction, the skill-guided model’s risk separation for individual compounds was worse than naive despite the higher overall AUC. DiffDock confidence scores yielded near-random VS performance (AUC 0.538 and 0.564 for naive and skill protocols, respectively). Although permutation tests confirmed that these values are statistically above chance (*P* ≤ 1/(*B* + 1) = 1.0 × 10^−4^ at *B* = 10,000 permutations), the practical effect was negligible (Cliff’s *d* = 0.08 and 0.13), with EFs below 1.0 at most thresholds, indicating no useful active enrichment for hit triage with either protocol.

### Computational cost

DiffDock dominated the total compute cost: approximately 182 GPU-hours, against approximately 13 GPU-hours for Uni-Dock (both arms combined for each engine). Expressed as throughput over the compounds actually scored, Uni-Dock processed roughly 1,110 compounds per GPU-hour (about 0.9 GPU-hours per 1,000 scored ligands, or ≈3.3 s per compound), against roughly 83 compounds per GPU-hour for DiffDock (about 12 GPU-hours per 1,000, or ≈44 s per compound), an approximately 13-fold throughput advantage for the physics-based engine (Table 4). These are per-engine averages over both arms; the per-arm wall-time was not separately logged in the deposited pipeline records, so Table 4 reports only per-engine figures and per-arm timing is not reconstructed to avoid mixing measured and apportioned numbers. Compute-throughput comparisons across engines should account for the fact that DiffDock’s wall-time includes the ESM-embedding pre-computation step in the skill-guided arm, which is amortized over inference but not over the per-compound headline figure.

**Table 4.** GPU wall-time and throughput per engine (naive + skill arms combined), on 2 NVIDIA RTX 4500 Ada GPUs. Throughput is computed over compounds actually scored (Uni-Dock 14,384; DiffDock 15,059).

Engine	Total GPU wall-time (h)	Compounds scored	Throughput (compounds/GPU-h)	GPU-h/1,000 scored	Mean time/compound (s)
Uni-Dock	≈13	14,384	≈1,110	≈0.9	≈3.3
DiffDock	≈182	15,059	≈83	≈12.1	≈43.5
Total	≈195	29,443	n/a	n/a	n/a

n/a, not applicable; GPU, graphics processing unit

### The effect of skill-guided protocol selection

The skill file primarily affected Uni-Dock protocols (Table 1), recommending a larger search box (25 versus 20 Å), higher exhaustiveness (32 versus 8), Meeko-based preparation (versus OpenBabel), and PAINS/Brenk pre-filtering. These changes yielded a statistically significant paired AUC improvement on the *n* = 4,303 compounds scored by both protocols (+0.020; 95% CI 0.007 to 0.034; paired bootstrap *P* = 0.002), at the cost of reduced top-of-list enrichment (EF@1% and EF@0.5%; see above). For DiffDock, the skill file’s influence was limited to library filtering (since DiffDock has no equivalent of box size or exhaustiveness parameters), and no significant paired AUC difference was observed (*n* = 4,765 compounds scored by both protocols; ΔAUC = +0.0075, DeLong *P* = 0.63).

## Discussion

On this single FPR2 case study, an LLM coding agent executed the full VS pipeline under human scientific supervision, and physics-based docking (Uni-Dock) materially out-ranked diffusion-based ML docking (DiffDock v1, native confidence score, under the tested configuration) on a 7TM GPCR target. The paired DeLong ΔAUC for Uni-Dock skill-versus-naive is +0.020 on the *n* = 4,303 intersection of compounds scored by both protocols (DeLong *P* = 3.4 × 10^−3^, paired BCa CI 0.007 to 0.034; [30]); this is the per-compound effect attributable to the joint Uni-Dock skill-versus-naive protocol shift, not to the skill-file mechanism in isolation (see “The skill-versus-naive contrast is multi-axis”, below). The +0.020 effect is statistically distinguishable from zero but small in absolute magnitude and is accompanied by a degradation in top-1% enrichment (EF@1% 2.67 → 1.23; EF@0.5% 3.33 → 0.00; data/metrics_all.csv). These 2 facts must be carried into the interpretation rather than smoothed over.

### Agentic AI for VS pipeline construction

The agent completed the entire pipeline from ChEMBL queries to statistical analysis in approximately 195 GPU-hours (≈182 h DiffDock, ≈13 h Uni-Dock; values reported as cluster wall-time, not independently logged in data) and required only high-level scientific direction from the researcher. The software-engineering load (database queries, file-format conversions, batch job management, score parsing, and statistical evaluation) was handled end-to-end by the agent. The researcher’s role shifted from writing code to setting scientific direction and reviewing the agent’s choices. This workflow is therefore more accurately described as agent-assisted orchestration under human scientific supervision than as autonomous scientific discovery: The investigator selected the target, designed the benchmark, authored the skill file, supervised the runs, and performed quality control, while the agent carried the software-engineering execution. The exact model snapshot, Claude Code CLI version, MCP/plugin set, and the full agent transcript are pinned in the “Methods” section and the Supplementary execution log, so this run is, in principle, reproducible from the deposited artifacts.

The skill file produced larger downstream changes in the Uni-Dock arm than in the DiffDock arm, although the 2 engines were each modulated on multiple axes. For Uni-Dock, the skill file changed receptor and ligand preparation tools, ligand 3D generation, chemical filters, docking box size, and exhaustiveness. For DiffDock, it changed the library (PAINS/Brenk filter), the number of denoising steps (20 → 25), the number of samples per complex (10 → 20), and the pre-computation of ESM embeddings. The DiffDock parameter changes are real but did not move the headline ranking metric: paired ΔAUC = +0.008, DeLong *P* = 0.63 (data/statistical_analysis_v2.json). The most parsimonious reading is that Uni-Dock’s parameters are more sensitive to the kinds of edit the skill file makes, not that DiffDock has fewer knobs in absolute terms; the skill file did exercise DiffDock’s user-configurable inference parameters, but those edits did not translate into improved ranking on this 7TM GPCR target under the tested configuration.

### The skill-versus-naive contrast is multi-axis, not a controlled skill-file ablation

The naive and skill-guided arms differ simultaneously on at least 5 dimensions: ligand-preparation tool, 3D conformer generation, PDBQT conversion path, PAINS/Brenk medicinal-chemistry filtering, and docking box/exhaustiveness settings. The +0.020 paired ΔAUC therefore cannot be attributed to the skill-file mechanism in isolation. Because the PAINS/Brenk medicinal-chemistry filter is applied only in the skill arm, much of the present FPR2 ΔAUC is plausibly a consequence of upstream library curation (a chemotype-restricted active set of 570/600 surviving validated actives in the Uni-Dock skill arm) rather than a generalizable docking-discrimination improvement; a factor-isolated ablation that re-prepares and re-docks the same PAINS/Brenk-filtered subset under both parameter settings would be needed to fully separate these contributions and is left to future work.

A partial control is, however, already available in the paired within-engine analysis. Because that comparison is computed on the intersection of compounds scored by both arms (Uni-Dock *n* = 4,309: 340 actives and 3,969 decoys; DiffDock *n* = 4,791: 533 actives and 4,258 decoys), it holds the evaluated compound set fixed and varies only the docking parameters and scoring; it is therefore not confounded by the filtering-induced shift in library composition that complicates the unpaired AUCs. On this fixed compound set, the Uni-Dock skill-versus-naive difference persists (paired AUC 0.717 → 0.738, DeLong *P* = 3.4 × 10^−3^) and the DiffDock difference remains nonsignificant (0.431 → 0.438, *P* = 0.63); these paired AUCs are computed on the common subset and differ from the full-set values in Table 3, which use each arm’s complete scored set. The Uni-Dock global-ranking gain is thus not merely an artifact of evaluating the skill arm on a smaller, chemotype-restricted active set. The residual limitation, which we do not overstate, is that 556 (Uni-Dock) and 410 (DiffDock) compounds retained by the skill filter were never scored under the naive parameters, so this fixed-set comparison covers the intersection rather than the entire filtered library, and filtering itself, being one of the interventions under study, cannot be fully unbundled from the other protocol changes.

The fact that the skill file was authored by the same investigator who curated the dataset and assessed the outcome introduces a further confound: A “skill-files-help” claim on this single configuration is not separable from “the investigator’s docking choices were well-matched to FPR2.” Breaking that circularity would require several controls absent here: a negative-control skill file engineered to make plausibly bad choices, skill files authored by external investigators, blinded evaluation of the 2 arms, and a pre-registered analysis plan. Until then, the present result is best read as *N* = 1 on every axis (one target, one engine pair, one skill-file instantiation, and one LLM snapshot) and is reported as a case study rather than a framework-level claim.

### Practical implications: ranking power versus early enrichment

The 2 Uni-Dock arms exemplify different failure modes when used to triage a screening library. Uni-Dock skill-guided improves *global* ranking discrimination (paired DeLong ΔAUC = +0.020, *P* = 3.4 × 10^−3^; Cliff’s *d* moves from 0.40 to 0.47) but *degrades* enrichment at the very top of the ranked list (EF@1% 2.67 → 1.23; EF@0.5% 3.33 → 0.00). For a practitioner whose downstream pipeline picks compounds from the very top of the list, the naive protocol is the more useful predictor on this dataset; for a workflow that uses the full ranking (e.g., as a feature in downstream re-ranking or active learning), the skill-guided protocol is preferable. The formal cross-target generalization question, which would require multiple targets evaluated under a common protocol contrast, is left to future work.

The continuous-NRI and IDI metrics computed on the intersection set are directionally consistent with this AUC-up/top-rank-down pattern, but must be read with 2 caveats. First, the NRI/IDI implementation in this work uses raw docking scores (Vina kcal/mol; DiffDock logits) without a calibrated score-to-probability mapping (Platt or isotonic), so they should be interpreted as ranking-shift statistics rather than as proper-scoring-rule improvements; cross-arm absolute magnitudes are not strictly comparable across engines on different score scales. Second, with those caveats, the within-Uni-Dock values (cNRI_total = +0.185, *z* = 4.65, *P* = 3.3 × 10^−6^; IDI = −0.040, *z* = −3.10, *P* = 1.9 × 10^−3^; data/statistical_analysis_v2.json) describe a mid-rank gain (more non-events demoted than events demoted) compensated by a top-rank loss (lower mean score on true actives under skill guidance), exactly as the EF profile shows.

### Physics-based versus ML-based docking on this GPCR

Uni-Dock achieved ROC AUC = 0.70 to 0.73 (medium effect, Cliff’s *d* = 0.40 to 0.47) while DiffDock v1 yielded near-chance ranking (AUC = 0.54 to 0.56; Cliff’s *d* = 0.08 to 0.13). The DeLong test on the intersection-scored set [30] gave ΔAUC = 0.23 to 0.29 for every Uni-Dock versus DiffDock pair, with 2-sided *P* values reported as *P* < 10^−15^ (Benjamini–Hochberg corrected over the family of 6 pairwise comparisons; raw values from the asymptotic DeLong implementation in data/statistical_analysis_v2.json range from 2.2 × 10^−43^ to 9.9 × 10^−79^, but tail probabilities at that magnitude exceed the resolution of the asymptotic normal approximation at *n* ≈ 5,000 to 10,000 and should be read as “below the resolvable floor of the test”). For FPR2, these AUCs sit within the range reported for retrospective GPCR VS benchmarks with structure-based scoring [32,33] (and for ultra-large library docking on G-protein-coupled and related targets [34]); we report them as a case study and not as evidence that physics-based docking generalizes favorably to all GPCR targets [11].

DiffDock’s confidence scores are statistically distinguishable from chance under permutation (*P* ≤ 1/(*B* + 1) = 1.0 × 10^−4^ at *B* = 10,000 permutations, the achievable floor of the test), but the practical utility for hit triage is negligible: EFs below or near 1.0 at the 1% to 10% thresholds (data/metrics_all.csv) indicate that, on this target, DiffDock v1 native confidence does not provide useful active enrichment for the very top of a ranked list. Independent benchmarks attribute this to the DiffDock training distribution: deep-learning docking methods can rely on near-neighbor cases in their training set for apparent generalization [7], and they fail on structurally novel targets [8,9], with further evidence that protein–ligand structure-based deep networks struggle to predict binding affinities outside their training distribution [35]. The present FPR2 result is therefore consistent with this broader picture rather than anomalous.

This scoping matters: The present benchmark evaluates the usefulness of DiffDock’s native confidence score for retrospective VS ranking, not diffusion docking as a general methodology. In contemporary practice, diffusion models are frequently used for pose generation with ranking delegated to a separate rescoring step, so the claim defended here is restricted to DiffDock v1, native confidence score, under the configuration tested. Rescoring DiffDock poses with Vina or with a learned scoring function such as GNINA [36] is a documented route to recover usable VS rankings from diffusion-generated pose ensembles [37], and DiffDock-L with its expanded training set [9] is a logical next configuration; neither was evaluated here and neither should be inferred from this paper’s negative DiffDock result.

The redocking results separate pose recovery from ranking power. Vina’s physics-based scoring reproduced the crystallographic pose to 0.22 to 0.39 Å (Table 2). Uni-Dock’s batch-mode redocking RMSD (5.2 to 5.7 Å) is markedly worse than its strong ranking performance, which is consistent with the general finding that pose-recovery accuracy and VS ranking power are only loosely correlated across docking workflows [25,32,38]. We refrain from attributing this to a single cause: Batch-mode preparation with a large search box, receptor preparation, binding-site definition, and ligand protonation or tautomer handling are all plausible contributors, and the present data do not separate them. DiffDock failed to reproduce the crystallographic binding pose under both protocols (RMSD 23.4 Å naive, 28.7 Å skill-guided), placing the predicted ligand entirely outside the orthosteric pocket. We previously flagged the under-representation of GPCRs in the PDBBind training distribution underlying DiffDock as a likely contributor to this failure; we leave that as a hypothesis here since we did not re-derive the GPCR fraction of PDBBind from primary sources for this revision. Together, the pose-recovery and VS results say only that DiffDock v1 native confidence is not a useful ranker on FPR2; they do not, on their own, identify a single cause.

### Limitations

This case study has several limitations. It examines a single target, FPR2, so results may differ for target classes well represented in DiffDock’s training distribution (e.g., kinases); generalization across targets and protein families is left to future work and should be established before any cross-target claim. The docking used DiffDock v1 with its native confidence score, so DiffDock-L [9] and learned-rescoring pipelines [36,37] are not evaluated. The reported BCa CIs quantify resampling uncertainty over compounds, not run-to-run variance over docking seeds: Stochastic ligand sampling in Vina-based engines is known to produce nontrivial single-replicate variation, and the present +0.020 paired ΔAUC was computed from a single seed per arm, so multi-seed replication (with a per-arm seed sweep and a paired across-seed comparison) is a needed next step before this magnitude is treated as deterministic. Because the skill file was authored by the same investigator who curated the dataset and assessed the outcome, and was not compared against a negative-control or external skill file, the present result is not a clean ablation of the skill-file mechanism. Downstream PAINS/Brenk filtering also removes a substantial fraction of validated actives from the skill arm (570 of 1,000 Uni-Dock skill-arm actives retained; 573 of 1,000 in the DiffDock skill arm; data/metrics_all.csv), so the skill-arm AUC is computed on a chemotype-restricted active set, a survivor-bias caveat to bear in mind when comparing skill-arm to naive-arm enrichment numbers. Finally, no prospective experimental validation of top-ranked hits was performed.

## Conclusion

In this single-target FPR2 case study, an LLM coding agent ran the full VS benchmark under author supervision, and a lightweight skill file produced a paired ΔAUC for Uni-Dock of +0.020 (95% BCa CI 0.007 to 0.034; DeLong *P* = 3.4 × 10^−3^; n_paired = 4,303 compounds), corresponding to an unpaired marginal shift from AUC = 0.701 (naive, *n* = 9,519) to AUC = 0.733 (skill, *n* = 4,865). The same skill-guided protocol degraded top-of-list enrichment on the same FPR2 ground truth: Unpaired EF@1% fell from 2.67 to 1.23 and unpaired EF@0.5% from 3.33 to 0.00; on the paired intersection ΔEF@1% = −3.24 (BCa CI −4.94 to −1.19, 2-sided *P* = 6.0 × 10^−4^). The net effect of skill-file guidance was a global-ranking gain coupled with a top-rank loss rather than a uniform improvement.

Three implications follow, all scoped to this FPR2 configuration and to the specific skill-file instantiation tested.

• Under author supervision, an LLM coding agent can carry the software-engineering burden of an end-to-end VS pipeline (library curation, preparation, docking, scoring, statistics, and figures), freeing the medicinal chemist to direct the science rather than the tooling.
• For FPR2, a 7TM GPCR that is under-represented in PDBBind, physics-based docking (Uni-Dock, AUC = 0.70 to 0.73) is the appropriate engine; DiffDock v1 with its native confidence score remained near random (AUC = 0.54 to 0.56) under the tested configuration and should not be used here for hit triage without rescoring or a GPCR-aware retraining.
• Structured skill files are a workable interface for injecting expert protocol decisions into agent-driven scientific pipelines, but the paired ΔAUC = +0.020 on FPR2 (Uni-Dock; the unpaired marginal estimate from per-arm scored sets is +0.032, 0.7333 versus 0.7014) is confounded with simultaneous changes in preparation tool, ligand generator, PDBQT conversion, PAINS/Brenk filtering, box definition, and exhaustiveness; a factor-isolated ablation, and a same-active-set re-evaluation that controls for the ~43% PAINS/Brenk attrition of validated actives in the skill arms (570 of 1,000 curated actives scored under Uni-Dock skill; 573 under DiffDock skill), are needed before the skill-file mechanism itself can be credited. Because each protocol was executed once with no explicit seed control, the +0.020 estimate is furthermore a single-run observation; multi-seed docking and repeated agent executions are required to characterize its variance before the magnitude is treated as stable rather than as partly reflecting stochastic docking variation.

## Data Availability

All code, data, and the skill file are archived on Zenodo under DOI 10.5281/zenodo.21083997 [17] (code under MIT license; data under CC BY 4.0). The deposit contains the skill file (SKILL.md), the 3 analysis scripts (compute_metrics_v2.py, statistical_analysis_v2.py, and generate_figures_v2.py), the frozen numerical outputs (per-protocol metrics, ROC and PR curves, and the statistical-analysis JSON), the main and supplementary figure files, and the complete pipeline archive vs_benchmark_v2.zip. To reproduce the analysis, unpack vs_benchmark_v2.zip into a working directory and run the 3 scripts in its scripts/ subdirectory in order; each script resolves its input paths relative to the unpacked tree, not to the deposit root. The same files are mirrored at https://github.com/oslo-medchem/VS-UniDock-vs-DiffDock; the exact git commit corresponding to this submission is tagged csbj-submission-r2, reachable at https://github.com/oslo-medchem/VS-UniDock-vs-DiffDock/tree/csbj-submission-r2.
