# Supplementary material for: Skill-File-Guided Virtual Screening: A Single-Target Formyl Peptide Receptor 2 Case Study of Large-Language-Model-Coordinated Physics and Diffusion Docking
Source: Comput Struct Biotechnol J. 2026 Sep 9;35(1):0187. doi: 10.34133/csbj.0187 (PMC13554421; doi:10.34133/csbj.0187)
Supplement: Supplementary file 1 [file csbj.0187.f1.zip › Figure_S2.pdf]

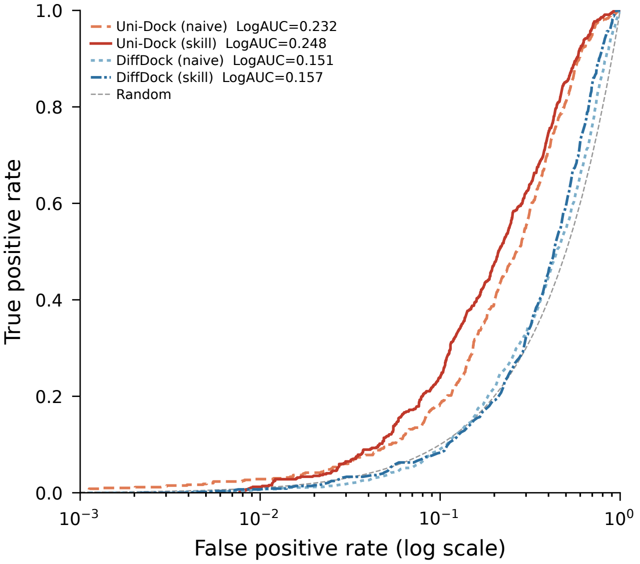
Uni-Dock (naive) LogAUC=0.232
Uni-Dock (skill) LogAUC=0.248
DiffDock (naive) LogAUC=0.151
DiffDock (skill) LogAUC=0.157
Random
True positive rate
False positive rate (log scale)
0.0
0.2
0.4
0.6
0.8
1.0
10^{-3}
10^{-2}
10^{-1}
10^{0}
