# Supplementary material for: Skill-File-Guided Virtual Screening: A Single-Target Formyl Peptide Receptor 2 Case Study of Large-Language-Model-Coordinated Physics and Diffusion Docking
Source: Comput Struct Biotechnol J. 2026 Sep 9;35(1):0187. doi: 10.34133/csbj.0187 (PMC13554421; doi:10.34133/csbj.0187)
Supplement: Supplementary file 1 [file csbj.0187.f1.zip › Figure_S3.pdf]

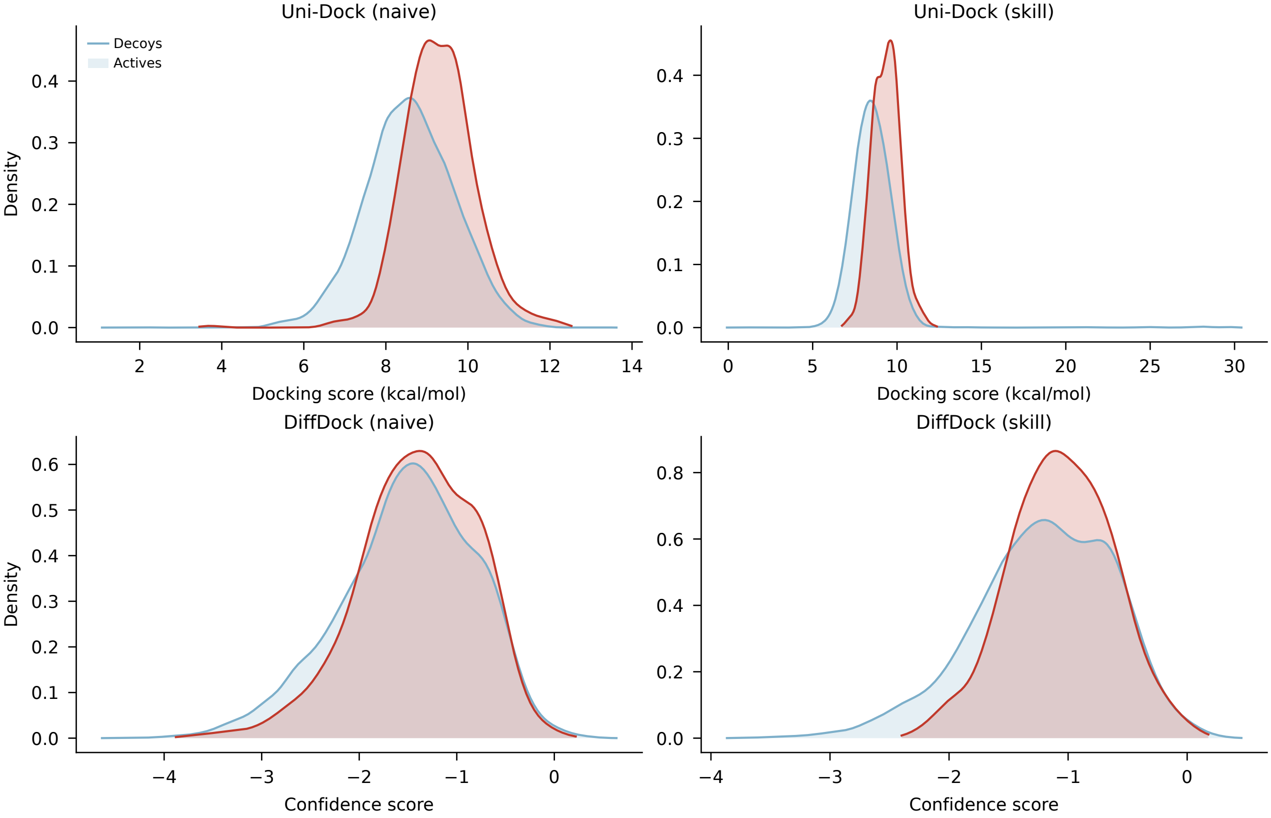

Uni-Dock (naive)
Uni-Dock (skill)
DiffDock (naive)
DiffDock (skill)
Decoys
Actives
Density
Docking score (kcal/mol)
Confidence score
