# Supplementary material for: Skill-File-Guided Virtual Screening: A Single-Target Formyl Peptide Receptor 2 Case Study of Large-Language-Model-Coordinated Physics and Diffusion Docking
Source: Comput Struct Biotechnol J. 2026 Sep 9;35(1):0187. doi: 10.34133/csbj.0187 (PMC13554421; doi:10.34133/csbj.0187)
Supplement: Supplementary file 1 [file csbj.0187.f1.zip › Figure_S4.pdf]

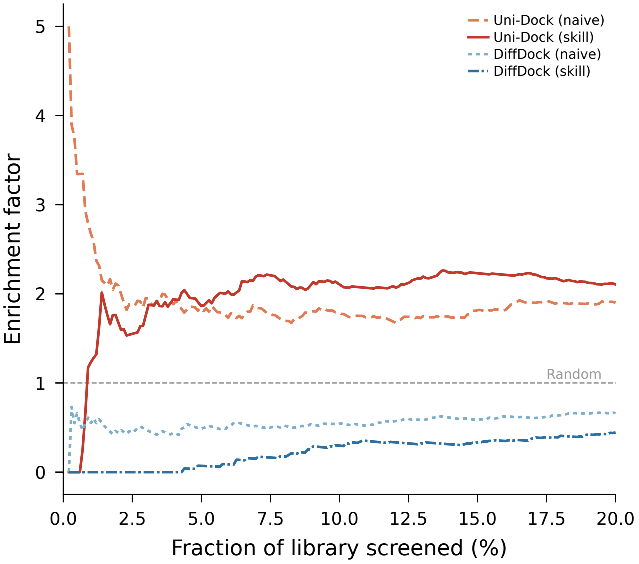

Uni-Dock (naive)
Uni-Dock (skill)
DiffDock (naive)
DiffDock (skill)
Random
Enrichment factor
Fraction of library screened (%)
0
1
2
3
4
5
0.0
2.5
5.0
7.5
10.0
12.5
15.0
17.5
20.0
