# Supplementary material for: Skill-File-Guided Virtual Screening: A Single-Target Formyl Peptide Receptor 2 Case Study of Large-Language-Model-Coordinated Physics and Diffusion Docking
Source: Comput Struct Biotechnol J. 2026 Sep 9;35(1):0187. doi: 10.34133/csbj.0187 (PMC13554421; doi:10.34133/csbj.0187)
Supplement: Supplementary file 1 [file csbj.0187.f1.zip › Figure_S5.pdf]

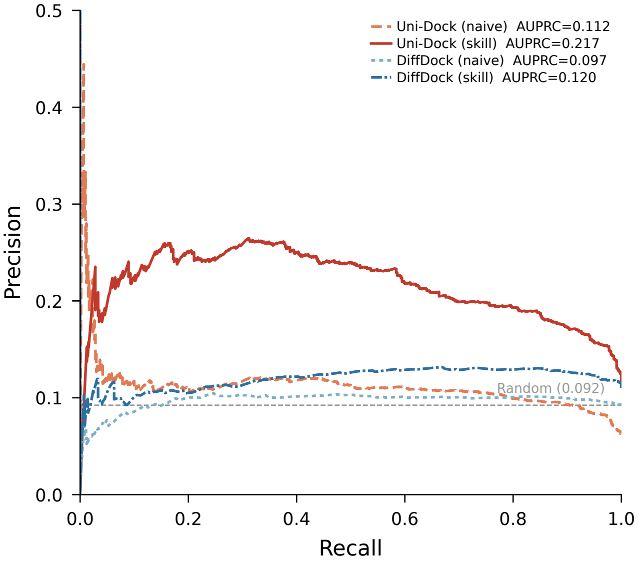

Uni-Dock (naive) AUPRC=0.112
Uni-Dock (skill) AUPRC=0.217
DiffDock (naive) AUPRC=0.097
DiffDock (skill) AUPRC=0.120
Random (0.092)
Precision
Recall
0.0
0.1
0.2
0.3
0.4
0.5
0.0
0.2
0.4
0.6
0.8
1.0
