# Supplementary material for: Skill-File-Guided Virtual Screening: A Single-Target Formyl Peptide Receptor 2 Case Study of Large-Language-Model-Coordinated Physics and Diffusion Docking
Source: Comput Struct Biotechnol J. 2026 Sep 9;35(1):0187. doi: 10.34133/csbj.0187 (PMC13554421; doi:10.34133/csbj.0187)
Supplement: Supplementary file 1 [file csbj.0187.f1.zip › Supplementary_Material_CSBJ_v2.docx]

**Supplementary Material**

*Skill-file-guided virtual screening: a single-target FPR2 case study of LLM-coordinated physics and diffusion docking*

**Osman Gani**

*Department of Pharmacy, Faculty of Mathematics and Natural Sciences, University of Oslo, Norway*

*Correspondence: osman.gani@farmasi.uio.no*

This document provides supplementary figures (S1–S5), supplementary tables (S1–S5), supplementary methods (S.1–S.4), and the agent execution log (S.5) supporting the main manuscript. All raw data, scoring matrices, and analysis scripts are deposited at the Zenodo record (DOI 10.5281/zenodo.21083997) and the GitHub repository referenced in the main text.

Supplementary Figures

Molecular property distributions for actives (orange) and decoys (blue). MW = molecular weight; LogP = calculated octanol–water partition coefficient; HBD/HBA = hydrogen-bond donors/acceptors; RotBonds = number of rotatable bonds; TPSA = topological polar surface area. The overlapping distributions confirm property-matched decoy generation (DUD-E principles, 10:1 decoy-to-active ratio).

**Fig. S1.** Molecular property distributions for actives (orange) and decoys (blue). MW = molecular weight; LogP = calculated octanol–water partition coefficient; HBD/HBA = hydrogen-bond donors/acceptors; RotBonds = number of rotatable bonds; TPSA = topological polar surface area. The overlapping distributions confirm property-matched decoy generation (DUD-E principles, 10:1 decoy-to-active ratio).

Semi-logarithmic ROC curves (log₁₀ FPR on the x-axis) for all four protocols. This representation emphasises early-enrichment performance, which is the regime most relevant to prospective virtual screening. The dashed diagonal indicates the random baseline (LogAUC ≈ 0.1446).

**Fig. S2.** Semi-logarithmic ROC curves (log₁₀ FPR on the x-axis) for all four protocols. This representation emphasises early-enrichment performance, which is the regime most relevant to prospective virtual screening. The dashed diagonal indicates the random baseline (LogAUC ≈ 0.1446).

Kernel density estimates of docking-score distributions for actives (solid lines) and decoys (dashed lines) across all four protocols. Greater separation between active and decoy curves indicates stronger discriminative power; the Uni-Dock protocols (top row) show clearer separation than DiffDock (bottom row), consistent with the AUC values in Table S1.

**Fig. S3.** Kernel density estimates of docking-score distributions for actives (solid lines) and decoys (dashed lines) across all four protocols. Greater separation between active and decoy curves indicates stronger discriminative power; the Uni-Dock protocols (top row) show clearer separation than DiffDock (bottom row), consistent with the AUC values in Table S1.

Enrichment factor (EF) curves as a function of the fraction of the ranked library screened (log scale). EF = (fraction of actives recovered) / (fraction of library screened). EF = 1 indicates random performance; values are reported at 0.5%, 1%, 2%, 5%, and 10% in Table S1.

**Fig. S4.** Enrichment factor (EF) curves as a function of the fraction of the ranked library screened (log scale). EF = (fraction of actives recovered) / (fraction of library screened). EF = 1 indicates random performance; values are reported at 0.5%, 1%, 2%, 5%, and 10% in Table S1.

Precision–recall curves for all four protocols. The dashed line indicates the baseline precision (active rate in the dataset, ≈ 9%). AUPRC (average precision) is reported in Table S1; the Uni-Dock skill protocol achieves the highest AUPRC (0.217) and the most favourable precision–recall trade-off in the high-recall regime.

**Fig. S5.** Precision–recall curves for all four protocols. The dashed line indicates the baseline precision (active rate in the dataset, ≈ 9%). AUPRC (average precision) is reported in Table S1; the Uni-Dock skill protocol achieves the highest AUPRC (0.217) and the most favourable precision–recall trade-off in the high-recall regime.

Supplementary Tables

**Table S1.** Comprehensive performance metrics for all four protocols. AUC = area under the receiver operating characteristic curve; BEDROC = Boltzmann-Enhanced Discrimination of ROC (Truchon & Bayly 2007); RIE = relative initial enhancement; EF = enrichment factor; LogAUC = semi-logarithmic AUC (Mysinger & Shoichet 2010); AUPRC = area under the precision–recall curve; pROC AUC = partial AUC in the indicated FPR range; MCC = Matthews correlation coefficient at the discriminative-optimal threshold. 95% confidence intervals are bias-corrected and accelerated (BCa) bootstrap (n = 10,000 iterations).

| **Metric** | **Uni-Dock naive** | **Uni-Dock skill** | **DiffDock naive** | **DiffDock skill** |
| --- | --- | --- | --- | --- |
| n (compounds / actives / decoys) | 9519 / 600 / undefined | 4865 / 570 / undefined | 9858 / 913 / undefined | 5201 / 573 / undefined |
| ROC AUC | 0.7014 | 0.7333 | 0.5384 | 0.5639 |
| AUC SE (Hanley–McNeil) | 0.0122 | 0.0124 | 0.0102 | 0.0131 |
| AUC 95% CI (BCa) | [0.677, 0.725] | [0.709, 0.758] | [0.518, 0.558] | [0.538, 0.590] |
| BEDROC (α=20) | 0.163 | 0.258 | 0.095 | 0.115 |
| BEDROC (α=80.5) | 0.143 | 0.173 | 0.058 | 0.090 |
| BEDROC (α=100) | 0.151 | 0.157 | 0.056 | 0.086 |
| RIE (α=80.5) | 2.253 | 1.476 | 0.630 | 0.816 |
| EF 0.5% | 3.33 | 0.00 | 0.44 | 0.35 |
| EF 1% | 2.67 | 1.23 | 0.77 | 0.87 |
| EF 2% | 2.08 | 1.67 | 0.60 | 0.79 |
| EF 5% | 1.83 | 1.86 | 0.74 | 0.91 |
| EF 10% | 1.78 | 2.11 | 0.91 | 0.84 |
| LogAUC (Mysinger) | 0.232 | 0.248 | 0.151 | 0.157 |
| AUPRC (avg. precision) | 0.112 | 0.217 | 0.097 | 0.120 |
| pROC AUC (0–1% FPR) | 0.506 | 0.498 | 0.499 | 0.499 |
| pROC AUC (0–5% FPR) | 0.514 | 0.513 | 0.495 | 0.498 |
| pROC AUC (0–10% FPR) | 0.524 | 0.535 | 0.494 | 0.497 |
| pROC AUC (0–20% FPR) | 0.552 | 0.582 | 0.498 | 0.497 |
| Recall @ 1% | 0.027 | 0.012 | 0.008 | 0.009 |
| Recall @ 5% | 0.092 | 0.093 | 0.037 | 0.045 |
| Recall @ 10% | 0.178 | 0.211 | 0.091 | 0.084 |
| Precision @ top 1% | 0.168 | 0.146 | 0.071 | 0.096 |
| MCC (optimal threshold) | 0.158 | 0.236 | 0.057 | 0.105 |

**Table S2.** Pairwise DeLong AUC comparisons (DeLong et al. 1988) with multiple-testing correction across all six pairs. Raw two-sided p-values are corrected by Bonferroni (family-wise α = 0.05; per-test α = 0.00833) and Benjamini–Hochberg false discovery rate (FDR α = 0.05). All five inter-engine comparisons remain significant after correction; the intra-engine Uni-Dock naive vs. skill comparison is significant under both corrections. The DiffDock naive vs. skill comparison is not significant under either correction.

| **Comparison (A vs. B)** | **AUC A** | **AUC B** | **ΔAUC** | **z** | **Raw p** | **Bonferroni p** | **BH-FDR p** |
| --- | --- | --- | --- | --- | --- | --- | --- |
| DiffDock naive vs. DiffDock skill | 0.4307 | 0.4383 | +0.0075 | 0.49 | 6.26e-1 | 1.00e+0 | 6.26e-1 |
| DiffDock naive vs. Uni-Dock naive | 0.4710 | 0.7031 | +0.2321 | 15.22 | 2.73e-52 | 1.64e-51 | 5.46e-52 |
| DiffDock naive vs. Uni-Dock skill | 0.4448 | 0.7345 | +0.2897 | 17.76 | 1.43e-70 | 8.58e-70 | 4.29e-70 |
| DiffDock skill vs. Uni-Dock naive | 0.4374 | 0.7045 | +0.2671 | 13.81 | 2.24e-43 | 1.34e-42 | 3.36e-43 |
| DiffDock skill vs. Uni-Dock skill | 0.4533 | 0.7340 | +0.2806 | 18.79 | 9.89e-79 | 5.93e-78 | 5.93e-78 |
| Uni-Dock naive vs. Uni-Dock skill | 0.7172 | 0.7376 | +0.0204 | 2.93 | 3.43e-3 | 2.06e-2 | 4.12e-3 |

**Table S3.** Paired BCa bootstrap of naive vs. skill protocols on the common-compound subset within each engine (n = 10,000 iterations). For each metric, Δ = skill − naive; positive values favour the skill-guided protocol. Two-sided p-values are derived from the bootstrap null distribution. Note that paired comparisons are conducted on common compounds (Uni-Dock n = 4,303; DiffDock n = 4,628) and therefore yield AUC values that differ slightly from the protocol-level values in Table S1, which use the full per-protocol library.

| **Engine** | **Metric** | **naive** | **skill** | **Δ (skill − naive)** | **95% CI (BCa)** | **p (two-sided)** |
| --- | --- | --- | --- | --- | --- | --- |
| Uni-Dock | AUC | 0.7172 | 0.7376 | +0.0204 | [0.007, 0.034] | 0.0024 |
| Uni-Dock | BEDROC (α=80.5) | 1.0000 | 1.0000 | +0.0000 | [-0.265, 0.000] | 0.0×10^0 |
| Uni-Dock | EF 1% | 4.1176 | 0.8824 | -3.2353 | [-4.942, -1.194] | 6.00e-4 |
| Uni-Dock | AUPRC | 0.1601 | 0.1521 | -0.0081 | [-0.030, 0.007] | 0.2962 |
| DiffDock | AUC | 0.4307 | 0.4383 | +0.0075 | [-0.023, 0.038] | 0.6304 |
| DiffDock | BEDROC (α=80.5) | 0.0762 | 0.0161 | -0.0601 | [-0.141, -0.002] | 0.0374 |
| DiffDock | EF 1% | 0.0000 | 0.0000 | +0.0000 | [0.000, 0.000] | 0.0×10^0 |
| DiffDock | AUPRC | 0.0903 | 0.0906 | +0.0003 | [-0.005, 0.005] | 0.9230 |

**Table S4.** Protocol parameters that differ between naive and skill-guided protocols. The skill-guided parameters are derived from the virtual-screening-pipeline skill file (≈ 400 lines) used to instruct the LLM coding assistant during protocol implementation. The DiffDock redocking caveat reflects the engine's YAML configuration loader overriding command-line arguments post-parse; this was corrected for the VS run with an explicit configuration file.

| **Parameter** | **Naive protocol** | **Skill-guided protocol** |
| --- | --- | --- |
| Uni-Dock: Receptor preparation | OpenBabel + Gasteiger charges | Meeko with explicit torsion handling |
| Uni-Dock: Ligand 3D generation | obabel --gen3D | RDKit ETKDGv3 + Meeko |
| Uni-Dock: Library filtering | None | PAINS + Brenk excluded |
| Uni-Dock: Docking box size | 20 Å (centred on FUI) | 25 Å (centred on FUI) |
| Uni-Dock: Exhaustiveness | 8 | 32 |
| DiffDock: SMILES input | Raw ChEMBL SMILES | RDKit canonical SMILES |
| DiffDock: samples_per_complex | 10 | 20 (VS); 10 (redocking, see caveat) |
| DiffDock: inference_steps | 20 | 25 (VS); 20 (redocking, see caveat) |
| DiffDock: ESM2 embeddings | Re-computed per run | Pre-computed (cached) |
| DiffDock: Confidence threshold | None | score > −0.5 |

**Table S5.** CONSORT-style compound-flow accounting across both protocols and both engines, addressing the reviewers’ request for an explicit attrition record from curated input through to scored compounds. Counts are reconciled against data/metrics_all.csv and 03_library_preparation_v2/skill/filter_report_v2.csv.

| **Stage** | **Naive protocol** | **Skill-guided protocol** |
| --- | --- | --- |
| Curated actives (from ChEMBL release 36 query, MaxMin / ECFP4, 302 Butina clusters) | 1,000 | 1,000 |
| Decoys generated (property-matched, Tanimoto < 0.35, FPR2-tested compounds excluded) | 9,829 | 9,829 |
| Input library before filtering | 10,829 | 10,829 |
| Compounds removed by PAINS + Brenk filter | 0 | 5,589 (incl. 427 actives, 5,162 decoys) |
| Library after filtering | 10,829 (1,000 actives + 9,829 decoys) | 5,240 (573 actives + 4,667 decoys) |
| Uni-Dock scored compounds (after ligand-preparation failures) | 9,519 (600 actives + 8,919 decoys) | 4,865 (570 actives + 4,295 decoys) |
| DiffDock scored compounds (after ligand-preparation failures) | 9,858 (913 actives + 8,945 decoys) | 5,201 (573 actives + 4,628 decoys) |
| Paired intersection used for DeLong + bootstrap (Uni-Dock) | 4,303 compounds (≈ 340 actives + 3,969 decoys) | (same set) |
| Paired intersection used for DeLong + bootstrap (DiffDock) | 4,765 compounds (≈ 533 actives + 4,258 decoys) | (same set) |

The 573-vs-570 discrepancy between DiffDock-skill and Uni-Dock-skill active counts (and analogous decoy discrepancies) reflects per-engine ligand-preparation attrition on an otherwise identical filtered list, not a separate filtering step. The paired n_pairs reported in the DeLong row (4,303 for Uni-Dock; 4,765 for DiffDock) differ from the NRI / IDI event-count totals (4,309 and 4,791) by 6 and 26 compounds respectively, reflecting tied-score exclusions in the Wilcoxon step that the NRI / IDI implementation does not apply.

Supplementary Methods

S.1 Software versions

Uni-Dock v1.1.3 (GPU-accelerated AutoDock Vina 1.2 scoring function); AutoDock Vina v1.2 (single-ligand redocking control); DiffDock v1.1 score-model checkpoint (workdir/v1.1/score_model) for both arms; ESM2-T33-650M for protein embeddings (pre-computed in the skill-guided arm); RDKit (ligand 3D embedding via ETKDGv3, fingerprint generation, MaxMin diversity picking); OpenBabel (naive protocol receptor and ligand preparation); Meeko 0.7.1 (skill protocol receptor and ligand preparation); scikit-learn (AUC, AUPRC); SciPy (Wilcoxon, Mann–Whitney, paired permutation); statsmodels (multiple-testing correction). The two conda environments (vina_dock, Python 3.12; diffdock, Python 3.9) are pinned in environment_vina.yml and environment_diffdock.yml in the Zenodo record (DOI 10.5281/zenodo.21083997); those YAML files are the authoritative source of version pins.

S.2 Active and decoy curation

Bioactivity records for FPR2 (CHEMBL4227) were retrieved via the chembl_webresource_client Python API from ChEMBL release 36 in March 2026. Records with pChEMBL ≥ 5.0, standard_relation = "=" (excluding qualified inequality values), and standard_type ∈ {EC50, IC50, Ki, Kd} were retained. Duplicate compound entries were resolved by InChIKey connectivity layer; when a compound carried multiple records, the highest pChEMBL value was retained. From this pool, 1,000 diverse actives were selected by Butina clustering (Tanimoto distance cutoff d = 0.4 on ECFP4 / Morgan radius 2, 2048-bit fingerprints) followed by MaxMin diversity picking. The Butina clustering yielded 302 clusters, providing scaffold diversity.

Decoys were generated from a drug-like ChEMBL pool (MW 150–600 Da, ALogP −2 to 5) by property-matched sampling: per active, candidates with MW ± 25 Da, LogP ± 1.0, HBD ± 1, HBA ± 2, and rotatable bonds ± 2 were eligible. Any compound with an FPR2 bioactivity record in ChEMBL (target_chembl_id = CHEMBL4227) was excluded from the decoy pool irrespective of measured value, to remove putative true binders from the negative class. Tanimoto ECFP4 similarity was constrained to < 0.35 against all actives (max Tc observed = 0.344). Formal charge was not matched explicitly. When the tolerance window yielded no matching pool compound for a given active, that active was skipped at the matching step. The 10:1 decoy-to-active ratio yields a standard error on ΔAUC of approximately 0.014 for ΔAUC ≈ 0.03 (Hanley–McNeil 1982).

S.3 Reproducibility

All analysis code, curated SMILES files, score matrices, and statistical scripts are deposited on Zenodo (DOI 10.5281/zenodo.21083997) and mirrored at the GitHub repository <https://github.com/oslo-medchem/VS-UniDock-vs-DiffDock> (tagged csbj-submission-r1 at the time of resubmission). The virtual-screening-pipeline skill file is included verbatim as SKILL.md at the top of the Zenodo record. Code is released under the MIT licence; data and figures under CC BY 4.0. Re-running the full v2 pipeline requires a CUDA-capable GPU (≥ 24 GB VRAM was used here for DiffDock with the skill-guided 20-samples / 25-steps configuration).

S.4 LLM use disclosure

The author used Claude Code (Anthropic, model claude-opus-4-8) as a coding assistant during pipeline development and analysis. The LLM was invoked with the virtual-screening-pipeline skill file (defining the skill-guided protocol) and an empty system prompt (defining the naive protocol). All scientific decisions, analyses, and manuscript content are the responsibility of the sole author. The LLM is not listed as an author, consistent with current authorship guidelines (ICMJE, COPE, 2024).

S.5 Agent execution log

The full agent transcript is deposited at the Zenodo record (DOI 10.5281/zenodo.21083997). The following summary captures the provenance items requested by the reviewers and not previously inferable from the deposited scripts.

**Model snapshot.** Claude Code, Anthropic, model identifier claude-opus-4-8.

**CLI and platform.** Claude Code CLI version 2.1.197. Ubuntu 24.04 LTS (Linux kernel 6.17.0-oem) on a workstation equipped with two NVIDIA RTX 4500 Ada GPUs (24 GB VRAM each), driver 580 series, CUDA 12.9 at the time of the runs.

**Run period.** Two pipeline executions, one per protocol, between 2026-03-01 and 2026-03-13; the deposited analysis outputs in data/statistical_analysis_v2.json and data/metrics_all.csv are timestamped 2026-03-13.

**Agent input.** Each run received a single natural-language instruction (“benchmark Uni-Dock vs DiffDock on FPR2 with 1,000 actives and property-matched decoys”). In the skill-guided arm the agent had read access to the virtual-screening-pipeline SKILL.md file at every planning step. No pre-written scripts were supplied at run start.

**Repetition.** The single-target FPR2 pipeline was executed once per protocol for this case study. No repeated runs were performed to characterise agent-to-agent or seed-to-seed variance; multi-run reproducibility on more targets is the subject of an in-preparation multi-target benchmark (reference [33] in the main text).

**Human interventions.** Human intervention during agent execution was limited to (i) the initial prompt, (ii) the supply or non-supply of the skill file, and (iii) restart of the agent on transient tool failures (network timeouts, GPU memory pressure) without scientific redirection. No manual code patches, no manual QC choices, and no scientific decisions were inserted into the run.

**Reproducibility scope.** Re-instantiation against the same source files (receptor PDB, ChEMBL release 36 records, skill file SHA-256 in the Zenodo deposit) is expected to reproduce the agent’s protocol choices qualitatively; quantitative reproducibility is bounded by stochastic ligand sampling in Vina-class engines (single-replicate seed dependence) and by ChEMBL release drift between query dates.
